# Neurenteric cyst of the craniovertebral junction treated to reduce recurrence using different strategies: Two case reports and a literature review

**DOI:** 10.1097/MD.0000000000033844

**Published:** 2023-06-09

**Authors:** Sue-Jee Park, Jong-Hwan Hong, Moon-Soo Han, Bong Ju Moon, Joo-Yeon Koo, Jung-Kil Lee

**Affiliations:** a Department of Neurosurgery Chonnam National University Hospital, Gwangju, Korea; b Department of Pathology, Chonnam National University Medical School and Research Institute of Medical Sciences, Gwangju, Korea.

**Keywords:** craniovertebral junction, instability, intraspinal cyst, neurenteric cyst, surgery

## Abstract

Neurenteric cysts are rare benign lesions that are usually located in the lower cervical and upper thoracic spine and extremely rare in the craniovertebral junction. It is generally challenging to completely remove the neurenteric cysts of the craniovertebral junction. We report the cases of 2 patients with neurenteric cyst in the ventral craniovertebral junction managed using different treatment strategies.

**Patient concerns:**

The first patient was a 64-year-old man. He man was admitted with headache, posterior neck pain, and a tingling sensation in both the forearms. The second patient was a 53-year-old woman. She was admitted with tingling sensations and numbness in both the hands and feet.

**Diagnoses:**

Cervical spine magnetic resonance imaging showed 2 intradural extramedullary cystic lesions in case 1 and a C2 to C3 intradural extramedullary cystic mass in case 2.

**Interventions and outcomes:**

The patient of the case 1 underwent a left C1 to C2 hemi-laminectomy and the cysts were completely removed. Eleven years after the surgery, there was no recurrence. In case 2, we performed a left C2 to C3 hemi-laminectomy and removed only a part of the outer membrane to enable sufficient communication with the surrounding normal subarachnoid space. After removing the cyst wall, the patient underwent C1 to 2 trans articular screw fixation to prevent cervical instability. Ten years after surgery, there was no recurrence of the cyst or new lesions.

**Lessons:**

Clinicians should consider neurenteric cyst in the differential diagnosis of arachnoid cyst or epidermoid cyst. If performing a complete surgical removal is difficult, partial surgical removal, using a cysto–subarachnoid shunt and stabilization, such as screw fixation, could be an alternative treatment option to reduce the risk of mortality and morbidity.

## 1. Introduction

Neurenteric cysts are rare benign lesions of the central nervous system, representing only 0.7% to 1.3% of all spinal cord tumors.^[1–3]^ They are classified as other malformities tumors and tumor-like lesions by the World Health Organization and are usually located at intradural intramedullary or extramedullary space in the lower cervical and upper thoracic spine; they are especially rare in the craniovertebral junction.^[4–6]^

A patient with a neurenteric cyst in the craniovertebral junction may present with symptoms of myelopathy, radiculopathy due to spinal cord or nerve root compression, recurrent meningitis, or pain.^[3,4]^ Complete surgical removal has been recommended as the treatment of choice to prevent recurrence.^[1,7]^ However, it is generally challenging to access and completely remove a neurenteric cyst at the craniovertebral junction.

We report the cases of 2 patients with neurenteric cysts in the ventral craniovertebral junction that were managed using different treatment strategies.

## 2. Case presentation

### 2.1. Patient 1

**Figure 1.**
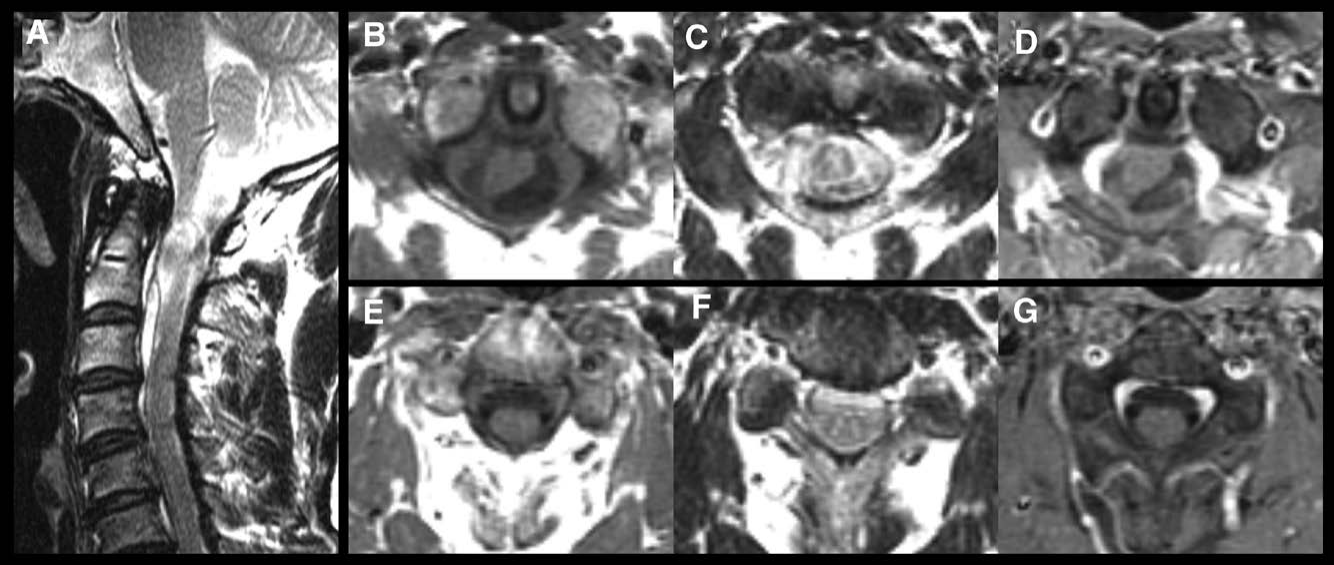
(Case 1) Preoperative spine magnetic resonance imaging (MRI) reveals (A) 2 cysts compressing the spinal cord. The lesions have isointense to low-intensity signals on T1-weighted MRI, high-intensity signal on T2-weighted MRI, and no enhancement after contrast-enhanced MRI at the level of C1–C2 (B–D) and C2–C3 (E–G).

A 64-year-old man was admitted to the hospital with headache, posterior neck pain, and tingling sensations in both forearms approximately 1 year ago; his symptoms began to worsen over a month. There were no neurological deficits, including gait disturbance, motor weakness, or sphincter dysfunction. Cervical spine magnetic resonance imaging (MRI) revealed 2 intradural extramedullary (IDEM) cystic mass lesions. The spinal cord at the C2 level showed poorly defined hyperintensity on T2-weighted images. One lesion was a 1.5-cm mass dorsal to the spinal cord from the craniovertebral junction to C1; the other was a 2.5-cm mass ventral to the spinal cord from C2 to C3 (Fig. 1A). These cystic lesions exhibited isointense to low-intensity signals on T1-weighted images (Fig. 1B and E) and high signal intensity on T2-weighted images (Fig. 1C and F). The mass was not enhanced after gadolinium administration (Fig. 1D and G).

We performed a left C1 to C2 hemi-laminectomy. intraoperatively, 2 white-colored cysts with thick membranes compressing the spinal cord and the right C2 nerve root were exposed. There was no connection between the 2 cysts, and they could be dissected away from the spinal cord and nerve root. The cysts were completely removed without injuring the surrounding neurovascular structures. Tissue was obtained from the cystic wall of the lesion at C2 to C3 for biopsy. After surgery, his symptoms improved, and he returned to daily life with reduced medication. The cysts were diagnosed as neurenteric cysts. Histopathological findings revealed that the tissue we thought was a cyst membrane was an inflammatory component, and the inner fluid was mucin-like material. Overall, the cyst was more likely to be a neurenteric cyst than an arachnoid cyst.

Postoperatively, cervical spine MRI revealed no residual or recurrent cystic masses. Follow-up cervical spine MRI performed 6 months after surgery revealed no recurrence; 11 years later, no recurrence was observed.

### 2.2. Patient 2

**Figure 2.**
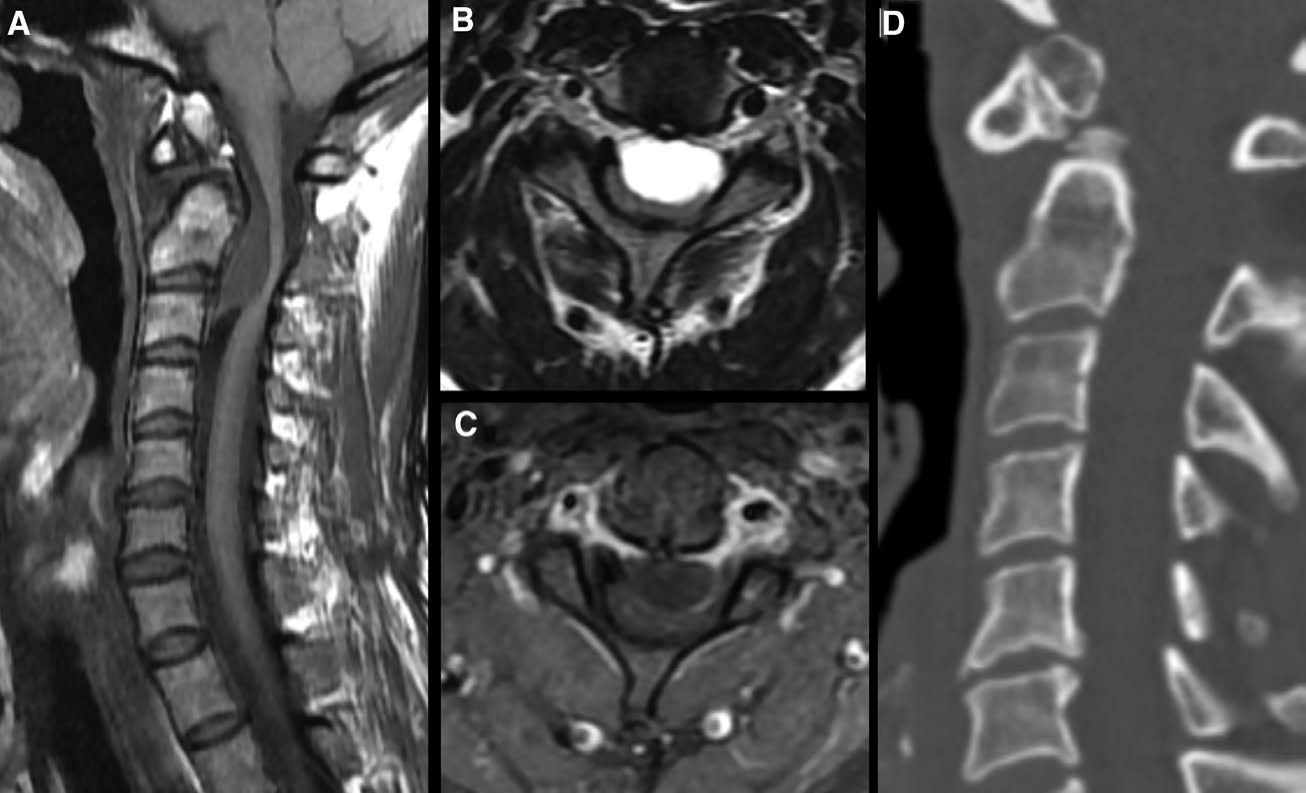
(Case 2) Preoperative spine magnetic resonance imaging (MRI) reveals a 30 mm × 15 mm mass at the level of C2–C3. The lesions have (A) an isointense to low-intensity signal on T1-weighted MRI, (B) high-intensity signal on T2-weighted MRI, (C) no enhancement after contrast-enhanced MRI, and (D) computed tomography sagittal slices show a circumferential corticated mass consistent with OS odontoideum.

**Figure 3.**
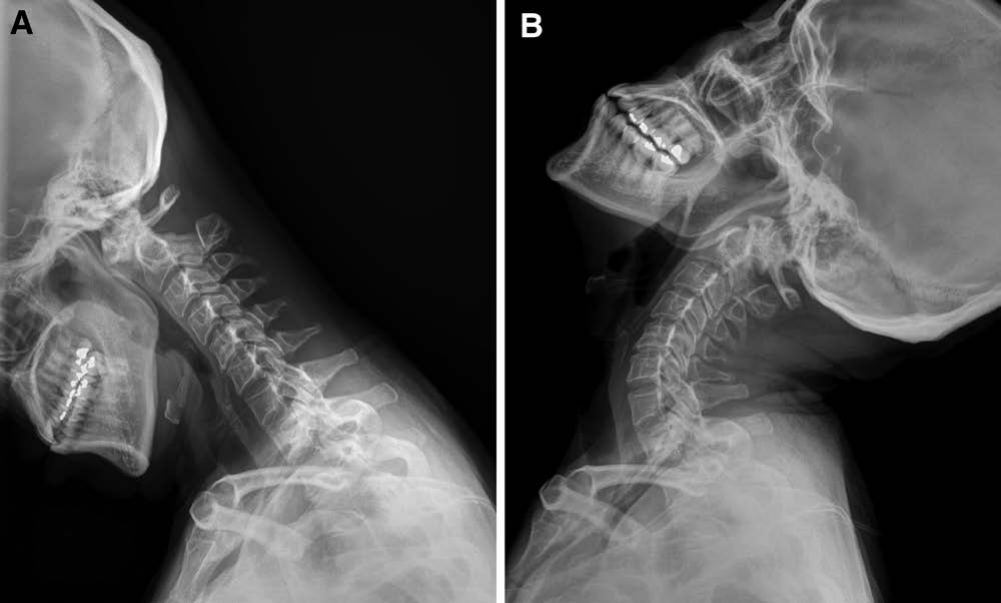
(Case 2) Plain dynamic flexion–extension radiographs show flexion (A) and extension (B) positions with subluxation on cervical flexion.

A 53-year-old woman presented with a 1 year history of tingling sensations and numbness in both hands and feet. She had no neurological deficits, including gait disturbance, motor weakness, or sphincter dysfunction. A C2 to C3 IDEM ventral spinal cord cystic mass was evident on cervical spine MRI scans, exhibiting isointense to low-intensity signals on T1-weighted images (Fig. 2A) and high signal intensity on T2-weighted images (Fig. 2B), with no enhancement after gadolinium administration (Fig. 2C). A sagittal CT image showed a circumferential corticated mass consistent with an OS odontoideum (Fig. 2D). Dynamic study images revealed cervical instability (Fig. 3A and B). The provisional diagnosis was an arachnoid cyst.

**Figure 4.**
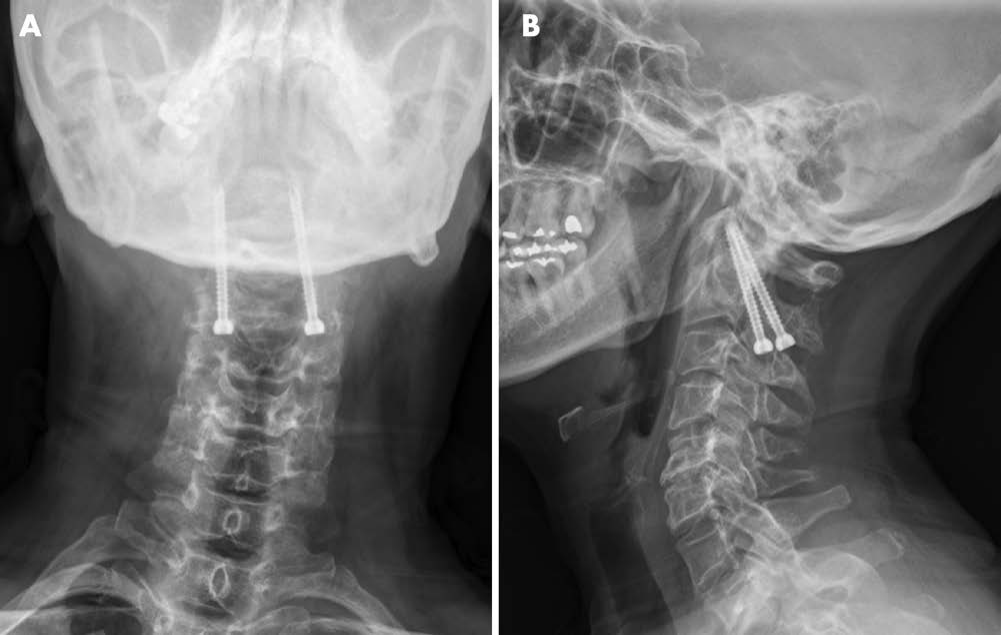
(Case 2) A postoperative plain lateral radiograph shows an intact alignment and implant.

We performed a left C2 to C3 hemi-laminectomy. Intraoperatively, white-colored cysts within a transparent gelatin-like material were found compressing the spinal cord. The transparent inner material of the cyst was easily removed using suction. Complete surgical resection of the cystic wall was difficult due to the spinal cord; therefore, we removed only part of the outer membrane to enable sufficient communication with the surrounding normal subarachnoid space. Biopsy of the cyst membrane was performed. After removing the cyst wall, we performed a C1 to 2 trans articular screw fixation to prevent cervical instability (Fig. 4). After surgery, the tingling sensation decreased in both hands.

**Figure 5.**
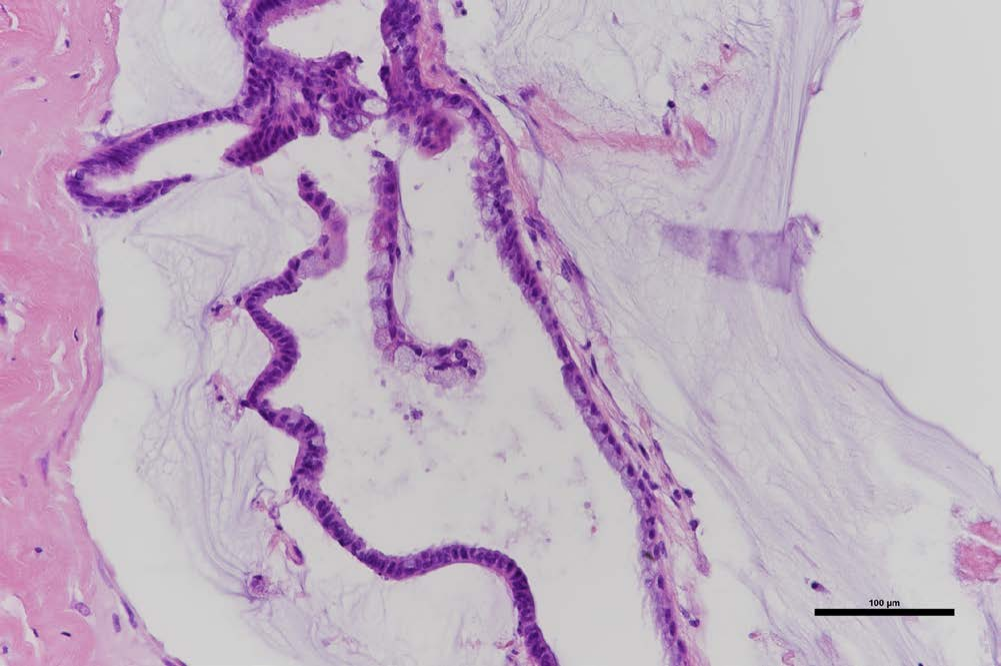
(Case 2) Histopathological findings of the resected cyst. Hematoxylin and eosin–stained photomicrographs show that the cyst wall is lined by simple columnar and cuboidal mucinous epithelium with isolated goblet cells.

Histological examination revealed that the mucin and cyst wall were lined by simple columnar and cuboidal epithelium with isolated goblet cells. Considering the overall features, the patient was diagnosed with a neurenteric cyst (Fig. 5). Ten years after the surgery, no recurrence was observed.

## 3. Discussion

Neurenteric cysts are rare benign lesions of the central nervous system. Over 90% of such lesions are IDEM lesions whose locations can cause pain, myelopathy and radiculopathy due to spinal cord and nerve root compression. These lesions are usually located ventral to the lower cervical and upper thoracic spinal cord but can occur rarely in the craniovertebral junction.^[4,7]^

Typical MRI findings of neurenteric cysts are isointense on T1-weighted images and hyperintense on T2-weighted images. However, they can appear differently on T1 weighted images, depending on protein content of the cysts. The cysts exhibited no contrast enhancement after gadolinium administration, however the cystic wall sometimes exhibited slight contrast enhancement. Cysts are mostly filled with fluid and have no solid component.^[3,8]^ Several theories have been suggested to explain their origin; the most widely accepted theory is that, during the embryogenic phase, abnormal communication between endodermal and ectodermal tissue is the main cause of cyst formation.^[5,7]^

Due to its embryological origin, the cyst wall pathologically resembles gastrointestinal and respiratory tissue. Hematoxylin-eosin staining showed that the cysts were lined by cuboidal or simple columnar epithelium with or without cilia containing frequent goblet cells, which produce secretions such as mucin. This secreted material, which it is thought to be the main cause of cyst growth, is mostly transparent and viscous or milky.^[3,5,8]^ In general, confirmation of cysts is based on pathological findings; however, we did not obtain any characteristic pathological tissues in case 1. In this case, other diseases, including arachnoid cysts, could be ruled out because the cyst contained sticky gelatin-like material and not cerebrospinal fluid, and the cyst contained inflammations. Based on these findings, we concluded that the diagnosis was neurenteric cyst.

Some articles have reported on patient symptoms, diagnoses, and surgical management to reduce recurrence; the cases of neurenteric cyst at the craniovertebral junction are summarized in Table 1. Until recently, complete surgical removal was considered the appropriate treatment; however, some studies have suggested that marsupialization is sufficient for most patients.^[1,8,9]^ Therefore treatment method was determined according to the operator’s clinical judgment without definitive guidance.

**Table 1:** Summarized surgically resected neurenteric cyst at the ventral craniovertebral junction.

Authors and Publication yr	Age/sex	Clinical symptom	Location of cyst	MRI features	Approach	Extent of resection	Follow-up	Recurrence
Breeze et al, 1990	37/M	Neck pain	Ventral	T1 hypointense	Suboccipital with C1–2 laminectomy	STR	1 yr	No
Koksel et al, 1990	40/M	Neck pain, quadriparesis	Ventral	T1 hypointense	Transoral	GTR	-	No
Fuse et al, 1998	49/M	Neck pain, headache	Ventral	T1/T2 slightly hyperintense	Suboccipital with C1 laminectomy	GTR	6 mo	No
	50/M	Neck pain, headache	Ventral	T1/T2 isointense	Transoral	GTR	-	No
Abe et al, 1999	60/F	Headache, neck movement limitation	Ventral	T1 isointense T2 hyperintense No enhancement	Far-lateral transcondylar	GTR	1 yr	No
Ergün et al, 2000	3/M	Neck pain, tetraparesis	Ventral	T1/T2 slightly hyperintense No enhancement	C3–C4 laminectomy, cysto–subarachnoid shunt after partial resection	STR	2 yr	No
Filho et al, 2001	30/F	Headache, nausea	Ventral to lateral	T1 hyperintense T2 heterogeneous No enhancement	Retro-sigmoid with C1 laminectomy	GTR	1 yr	No
	30/F	General seizure, ataxia, dysarthria	dorsal	T1 hyperintense	Suboccipital with C1 laminectomy	GTR	3 yr	No
	25/M	Headache	Ventral	T1 hyperintense Irregular enhancement	Retro-sigmoid with C1 hemi-laminectomy	STR	2 yr	No
de Oliveira et al, 2005	1/F	Recurrent meningitis	ventral	T1 hypointense T2 hyperintense No enhancement	Post-fossa (first) Transoral (second)	STR GTR	3 yr	Yes No
Liu and Couldwell, 2005	46/F	Pain in right shoulder, right ear, and interscapular area	Lateral	T1 isointense T2 hyperintense No enhancement	Far-lateral transcondylar	GTR	-	No
	26/F	Headache	Ventral to lateral	T1 hyperintense T2 slightly hyperintense Minimally enhancement	Far-lateral transcondylar	GTR	-	No
Menezes and Traynelis, 2006	8/F	Recurrent meningitis	Ventral	T1 hyperintense	Far-lateral transcondylar	GTR	13 yr	No
	30/M	Neck pain, Urinary incontinence, weakness of the left arm and both legs	Ventral	T1 hyperintense T2 hyperintense No enhancement	Far-lateral transcondylar	GTR	8 yr	No
	14/M	Headache, neck pain, quadriparesis	Ventral	-	Far-lateral transcondylar	GTR	4 yr	No
Clare et al, 2006	57/F	Neck pain with occipital and supraspinatus discomfort	Dorsal	T1 hypointense T2 hyperintense Marginal enhancement	Posterior fossa craniectomy with C 1 hemi-laminectomy	STR	-	No
Sakata et al, 2008	1/M	Tetraparesis	Ventral	T1 isointense T2 isointense No enhancement	Suboccipital with C1 hemi-laminectomy	GTR	2 yr	No
Shi et al, 2010	43/M	Neck pain, dysesthesia in both hands	Ventral	T1 hypointense T2 hyperintense No enhancement	Far-lateral transcondylar	GTR	3 yr	No
Shetty et al, 2013	7/F	Neck pain	Ventral to lateral	T1 hypointense T2 hyperintense Posterior wall enhancement	C1, 2 hemi-laminectomy (first) Far-lateral app. (second)	STR GTR	6 mo/-	Yes No
	36/F	Intermittent headache Right CN 6, 7, 8^th^ nerve palsy with cerebellar ataxia	Ventral to lateral	T1 isointense T2 Mixed density Posterior wall enhancement	Far-lateral app.	GTR	-	No
Menendez et al, 2019	24/F	Neck pain, left hemiparesis	Ventral	T1 hyperintense T2 hypointense DWI/ADC No restricted No enhancement	Minimal suboccipital craniectomy and left C1-C2 hemi-laminectomy	GTR	6 mo	No
Ozalp et al, 2019	16/M	Suboccipital pain, upper extremity weakness, Swallowing difficulty, Urinary incontinence	Ventral	T1 isointense T2 hyperintense	C 1, 2 laminectomy	GTR	3 mo	No
Sahoo et al, 2020	0/F	Upper limb weakness	Ventral	T1 hypointense T2 hyperintense	Suboccipital with C 1–3 hemi-laminectomy	GTR	-	No
Haque et al, 2020	11/M	Neck pain, quadriparesis	Ventral	T1 iso-hypointense T2 hyperintense	Suboccipital craniotomy with C 1 laminectomy	GTR	2.5 yr	No
Kim et al, 2021	16/F	Headache	Ventral	T1 hyperintense T2 hyperintense No enhancement	Far-lateral transcondylar	GTR	6 mo	No
Present case	64/M	Headache, post neck pain, both forearm tingling sense	Ventral	T1 iso-hypointense T2 hyperintense No enhancement	C 1, 2 hemi-laminectomy	GTR	11 yr	No
	53/F	Both hand and leg numbness	Ventral	T1 iso-hypointense T2 hyperintense No enhancement	C 2,3 hemi-laminectomy	STR	10 yr	No

- = not available, F = female, GTR = gross total resection, M = male, MRI = magnetic resonance imaging, STR = subtotal resection.

Clinicians should perform complete surgical removal if possible. However, complete removal of a neurenteric cyst in the craniovertebral junction is difficult; many important structures are located in the craniovertebral junction, including the vertebral artery and the lower cranial nerve. Moreover, the surgical field is usually narrow, because the amount of bone exposure should be tailored to avoid unnecessary bone drilling and reduce craniovertebral instability; retraction of the cerebellum, brainstem, and spinal cord should also be minimized to prevent neurological injury.^[10,11]^ Therefore, we performed partial surgical removal using a cysto–subarachnoid shunt with posterior screw fixation to prevent recurrence.

The generally accepted hypothesis is that some inflammatory/immune-response mediators initiate mucin hypersecretion in the respiratory system and gastrointestinal tract by activating a secretory cascade that results in the rapid release of mucin from the secretory granules in goblet cells.^[12,13]^ Based on this, we assumed that instability is a factor that triggers inflammatory reactions. Instability causes mucin secretion to become more active, which increases the size of the cysts as goblet cells secrete mucin. Therefore, in Case 2, we thought that the cyst would not recur if mucin secretion was reduced and the cyst wall was sufficiently removed so that the remnant cyst wall did not block communication. For these reasons, we suggest that partial removal of a cyst and stabilization through screw fixation are sufficient treatments to eliminate the cause of the cyst growth.

Partial removal and stabilization are an alternative treatment option for a neurenteric cyst in the craniovertebral junction, where complete surgical removal is difficult, and are thought to reduce the risk of mortality and morbidity. Although no recurrence was observed in case 2 during the 10-year follow-up period, the cyst could not be considered completely cured. More cases of these rare cysts, exploring other treatment options, should be reported to help define evidence-based recommendations for patients with neurenteric cysts in the craniovertebral junction.

## 4. Conclusion

Neurenteric cysts are rare benign cysts; until recently, complete surgical removal has been considered an effective treatment for reducing the risk for recurrence after surgery. However, it is often difficult to approach and completely remove neurenteric cyst surgically. We suggest that partial surgical removal, using a cysto–subarachnoid shunt and stabilization, such as screw fixation, could be an alternative treatment option for patients for whom complete surgical removal is difficult or whose cysts have severe adhesion to the surrounding neurovascular structures.

## Author contributions

**Conceptualization:** Jung-Kil Lee.

**Data curation:** Sue-Jee Park, Jong-Hwan Hong, Bong Ju Moon, Joo-Yeon Koo.

**Methodology:** Jung-Kil Lee, Moon-Soo Han.

**Software:** Sue-Jee Park.

**Supervision:** Jung-Kil Lee.

**Writing – original draft:** Sue-Jee Park, Moon-Soo Han, Joo-Yeon Koo.

**Writing – review & editing:** Sue-Jee Park, Jung-Kil Lee, Moon-Soo Han.
